# A Qualitative Study of Practitioners’ Views on Family Involvement in Treatment Process of Adolescent Internet Addiction

**DOI:** 10.3390/ijerph18010086

**Published:** 2020-12-24

**Authors:** Camilla Kin Ming Lo, Lu Yu, Yuet Wing Cho, Ko Ling Chan

**Affiliations:** Department of Applied Social Sciences, The Hong Kong Polytechnic University, Hong Kong, China; camilla.lo@polyu.edu.hk (C.K.M.L.); lu.yu@polyu.edu.hk (L.Y.); yuet-wing.cho@polyu.edu.hk (Y.W.C.)

**Keywords:** internet addiction, gaming disorder, treatment, family involvement

## Abstract

Despite emerging evidence of the effectiveness of a family-focused approach as an Internet addiction (IA) treatment modality for adolescents, little research has been done to explore family involvement in the treatment process from the clinician’s perspective. This study employed a qualitative design to examine practitioners’ views pertaining to the roles and challenges of family participation in IA intervention. In total, 10 practitioners working with adolescents with IA were interviewed. Thematic analysis was used to analyze the transcribed interviews. Three overreaching themes were synthesized: That family involvement in IA intervention is challenging yet important; shifting the focus from the adolescent to the relationship; and provision of individualized services and intervention to address the heterogeneous nature of cases. The findings show that family participation in IA treatment is successful in enhancing positive outcomes. The needs of adolescents with IA and family members are addressed through individual counseling and psychoeducation, respectively. Conjoint therapy sessions foster effective communication, improve family interactions and functioning, and restore relationships. However, caution regarding family dynamics is required when considering whether family involvement is appropriate. Practitioners need to establish therapeutic alliances and be flexible when working with family members in terms of the degree and arrangement of participation.

## 1. Introduction

A dysfunctional pattern of excessive Internet use and gaming has been one of the most concerning adolescent health problems in recent years. Owing to the lack of consensus on its definition and conceptualization, such dysfunctional Internet use and gaming patterns have been referred to by different interchangeable terms in previous literature. Most frequently, it has been termed Internet Addiction (IA), which is characterized by one’s inability to control the use of the Internet, leading to negative consequences in physical and psychological health [1]. IA has been used to describe a generalized pattern of addictive Internet use. More recently, gaming disorder, a specific form of Internet addiction, has been formally recognized by the World Health Organization [2] as a legitimate health diagnosis. In a recently published meta-analytic review of 113 epidemiologic studies, it was reported that the prevalence rates of IA and gaming disorder were 7.02% and 2.47%, respectively [3]. Research findings have consistently shown a range of adverse effects of IA on health and relational problems, including depression, sleep problems, and physical aggression [4]. Furthermore, a meta-analysis revealed a significant association between excessive Internet use and cognitive deficits [5].

In light of the emerging prevalence of IA, research exploring its correlates has proliferated. In particular, there is a burgeoning amount of research focusing on family influence on adolescents’ IA behaviors. A recent systematic review revealed that parent–child and family-related factors are consistently associated with IA among adolescents [6]. In terms of parenting, maladaptive parenting, including indulgence, overinvolvement, punitive disciplinary methods, frequent rejection, and low levels of warmth are related to the occurrence of adolescents’ problematic Internet use [7,8]. On the contrary, strong parental emotional support and care protect against IA [8,9,10]. Apart from parenting, constructive family functioning, including family roles and affective family involvement, is another prominent factor that contributes to lower IA risk among adolescents [11,12]. Family cohesion, as illustrated by a healthy parental relationship [13], good parent–child communication [14,15], and less conflict [10,12] have also been shown to have profound protective effects on the incidence and remission of IA in adolescents. In sum, these studies have shed light on the importance of adopting a family-based approach in treatment programs for adolescent IA.

Despite the large body of research that clearly demonstrates the association between family environment and adolescents’ IA, there is surprisingly only a dearth of studies adopting a family-focused approach as an IA treatment modality. Among the scant literature published in the field, Zhong et al. [16] suggested that family-based intervention was more effective in reducing Internet use compared to conventional individual IA intervention among the adolescent population, owing to its lasting treatment effects. Another study evaluated a brief three-week family therapy intervention program, in which parent–adolescent dyads engaged in activities to improve family cohesion, and found improvement in adolescents’ online gaming time and addiction severity [17]. A more recent six-week, multi-family group therapy intervention was also found to be effective in reducing adolescents’ IA behaviors [18]. The outcomes were partly explained by the strengthened positive parent–adolescent communication and closeness and adolescents’ satisfaction of psychological needs in real life. These studies provide initial and important evidence of the effectiveness of family-based intervention on alleviating adolescent IA through modifying familial risk and protective factors, such as family cohesion and parent–adolescent communication.

While the literature calls for additional evidence of family-based interventions using a rigorous, randomized, controlled study design to inform the evidence base of IA intervention, it is equally important to consider clinical expertise and patient characteristics, which are two of the three pillars of the foundation of evidence-based practice [19]. Currently, qualitative studies pertaining to clinicians’ experience in treatment processes of IA are very limited. The most relevant published research gathered clinicians’ views about IA treatment, some of which pointed to the importance of involving family members in treatment, highlighting family members’ roles in providing helpful information about the impacts of IA on the concerned individual and in taking part in evaluating treatment outcomes [20].

Before further studying the effectiveness of family-based intervention for IA, a crucial step is to clarify the precise role of family members in treatment and to understand whether particular adolescent–family dyads may require special care in treatment. Hence, the present qualitative study aimed to capture and analyze the experiences and challenges surrounding family involvement in IA treatment with adolescents from the practitioners’ perspective to better inform the design of future intervention and clinical practice.

## 2. Materials and Methods

### 2.1. Study Design and Sample

To recruit practitioners in the IA field, invitation letters were sent to specialized addiction counseling services, and family and youth social service centers in Hong Kong, and through the professional network of the first author. The participants were required to (1) be social workers/counselors/psychologists, (2) have at least one year of full-time experience working with adolescents with Internet addiction as part of their caseload, and (3) provide written consent. Eligible participants who met all the criteria participated in an in-depth individual interview that lasted approximately 45 min via Skype or Zoom. The interviews were conducted in Chinese (Cantonese) by a trained research assistant. A semi-structured interview guide was used to outline the flow of the interviews. Each participant received an HKD100 (approximately USD12.8) supermarket voucher as appreciation for participation. All interviews were conducted between 28 March and 11 July 2020.

The present qualitative study collected and analyzed practitioners’ views and practices regarding family involvement in IA intervention through in-depth interviews. Interviews were conducted based on the following discussion areas: (1) the subjective perspectives of practitioners on the roles of family members and the perceived effectiveness of family involvement in IA treatment, and (2) the experiences, difficulties, and considerations concerning family participation in intervention programs. Sample interview questions covered in each discussion area are listed in Table 1.

A total of 10 practitioners were recruited and interviewed in this study. Table 2 shows the characteristics of the participants. The participants were addiction counselors, social workers, and a counseling psychologist at specialized addiction treatment centers, and social workers in school and family settings. Eight participants specialized in providing addiction counseling services, while two specialized in working with youths and families. They had an average of 9.4 years of clinical experience and an average of 5.9 years of experience working with IA. Half of the participants were female. Their self-identified theoretical and intervention orientation included expressive art therapy, cognitive behavioral therapy, and narrative therapy.

### 2.2. Ethics Approval

The research project and protocol of the study was approved by the Human Subjects Ethics sub-committee of the Hong Kong Polytechnic University (Reference number HSEARS20190530002). A written consent form was signed by all participants, ensuring confidentiality and protecting the right to withdraw at any time during the study. No personal identifiers were collected from the participants during the interviews.

### 2.3. Data Analysis

All interviews were audiotaped, transcribed verbatim using a word processor, and coded using NVivo software for qualitative analysis. To describe and analyze the gathered information in an accessible format, a thematic analysis was performed [21]. Thematic analysis not only allows summarization of the actual data in a condensed and systematic format, but also generation of themes for pattern analysis and interpretation from the raw qualitative data [22]. It was used to identify patterns of themes within and across data in relation to participants’ experiences, views, and perspectives, and practices of involving family members in IA treatment and intervention. The first author read all the transcripts for familiarization with the data. The first author and two other coders (third author and a research assistant) independently coded one transcript, then discussed and resolved discrepancies in the coding. A codebook was then developed for coding subsequent transcripts and the coders were trained to code the data in accordance with the codebook. Considering one of the critical assumptions of thematic analysis that a flexible, organic, and reflexive approach should be adopted to guide the coding process, multiple coders and inter-rater reliability are not recommended [23]. Instead, after the two coders completed coding all the transcripts, the first author reviewed the coding and had a discussion with the coders to resolve discrepancies in the coding to ensure the rigor of data and the consistency of the analytical procedure. The active and reflexive coding process highlighted the subjectivity of the researchers and encouraged a more complex and nuanced theme development in the later stages of analysis. The high-frequency codes (mentioned by five or more participants) were then identified and conceptualized into meaningful themes at a latent level, aiming at drawing relationships between codes and capturing the implicit meanings of family involvement in IA treatment in the socially constructed experiences of the participants [21]. Table 3 delineates the phases of thematic analysis adopted from Braun and Clarke [21] and followed by the present study.

## 3. Results

Three major themes were constructed after a thorough analysis of the qualitative data collected from the interviews: (1) Family involvement in IA intervention is challenging yet important, (2) shifting the focus from the adolescent to the relationship, (3) provision of individualized services and intervention to address the heterogeneous nature of cases. A summarized outline of the themes with their respective illustrative quotes is provided in Table 4.

Theme 1: Family involvement in IA intervention is challenging yet important (*n* = 8)

Eight practitioners agreed that family involvement was effective in promoting the treatment process and enhancing positive outcomes. Specifically, it was of paramount importance for family members to understand the underlying needs of the clients and to empathize with them (*n* = 10). Practitioners engaged with family members, encouraging them to actively reflect on the clients’ IA behaviors and gain new perspectives on the latent functions of their Internet use. This was exemplified in the quote below:

*Parents are usually unaware of the unfulfilled needs and problems faced by their children. My strategy is to help them to understand the underlying needs of the IA behaviors and view the problem from the children’s perspective.*

(Participant 8)

Oftentimes, family members had unrealistic expectations of the clients regarding their change in IA behaviors, in the sense that their fixation on the problem impeded them from realizing the clients’ potential. Therefore, practitioners also worked with parents to adjust their expectations on parental roles and responsibilities (*n* = 5). The quote below was an example of how this was described in the interview:

*Internet abstinence is not tenable for adolescents. Therefore, I coach parents on how to adjust their expectations and to negotiate with their children to find a common ground where both parties find an acceptable and satisfactory balance.*

(Participant 4)

Another essential element was parent psychoeducation (*n* = 7). Practitioners aimed to equip family members with knowledge of IA, positive parenting approaches, effective communication skills, and conflict resolution strategies. The quote below showed how practitioners delivered psychoeducation to parents:

*Apart from working with adolescents, my work focuses heavily on parent education. I assist parents to review their parent–child relationship and their perceptions of parenting. I also share different parenting strategies with them through case study and role-play.*

(Participant 6)

Despite its importance, family involvement was fraught with different challenges. One of the practitioners explained how family members’ obliviousness of their personal contribution to the clients’ IA might serve as a potential obstacle for the IA treatment (*n* = 5):

*Some cases are tough to handle because parents become defensive by marginalizing themselves from the situation. It is difficult for them to accept that their personal history may contribute to their children’s IA .*

(Participant 5)

The effectiveness of family involvement was also circumscribed by family members’ frustration, stress, and helplessness (*n* = 6), coupled with complicated and ingrained family issues (*n* = 5):

*It is not unusual that parents, especially mothers, suffer from depression because of their children’s IA. Limiting the degree of engagement with family members might be a better option in these situations.*

(Participant 10)

Theme 2: Shifting the focus from the adolescent to the relationship (*n* = 6)

When family members were committed to the IA treatment, practitioners adopted a relationship-focused intervention orientation, as opposed to an individual-focused approach (*n* = 6). They created a safe and open space for parents and clients to process their thoughts and feelings rationally to foster mutual understanding and restore the parent–child relationship (*n* = 6). This two-way communication is illustrated by the following quote:

*We usually conduct a joint interview, which allows parents and their children to give voice to their hidden thoughts and emotions. Cohesive and effective communication is achieved so that they can move forward with conflict resolution and reconciliation, with my presence as a mediator.*

(Participant 6)

The foundation of the relationship-building capability with clients and family members was therapeutic alliances (*n* = 5). The practitioner in the quote below explained how a non-judgmental attitude and unconditional positive regard played a critical role in establishing trust and rapport:

*I believe the first step in building therapeutic relationships is to be non-judgmental and gain trust from the clients. Once they feel they are accepted and heard, they are more likely to engage in the process, take my advice, and make behavioral change.*

(Participant 1)

In contrast, family members questioned the professionalism and credibility of the practitioners when trust was limited in early relationship-building (*n* = 5). For example:

*Some parents have doubts about my approach to proceeding with the counseling sessions. They challenge my expertise as a social worker to mediate their family relationship.*

(Participants 2 and 10 )

Theme 3: Provision of individualized services and intervention to address the heterogeneous nature of cases (*n* = 8)

The majority of the practitioners were flexible in their therapeutic orientations, hinging on the clients’ age and gender, as well as their needs and motives (*n* = 8). Combinations of different approaches, ranging from cognitive behavioral therapy and motivational interviewing, to narrative, solution-focused, and expressive art therapy, were incorporated throughout the process. Similarly, intervention goals and the criteria to terminate the treatment were customized for each client. As a result, the clients demonstrated a myriad of outcomes over the course of varying treatment durations (*n* = 8).

While the importance of family involvement was highlighted in the aforementioned theme, practitioners considered family environment, parents’ willingness and motivation, and intervention goals, before deciding whether involving family members would be beneficial to the process (*n* = 8). For instance, the following quote was an example of how dysfunctional family dynamics and relationships would preclude parents from taking part in the intervention (*n* = 7):

*When the parent–child relationship is hostile and inefficacious, it is not suitable to invite parents to join the session, as arguments will potentially arise in the process. They are not mentally prepared to listen to one another and discuss the issue.*

(Participants 6 and 7 )

In light of the heterogeneity of clients’ background and treatment needs, the degree and arrangement of family involvement were determined on a case-by-case basis (*n* = 8). For instance, practitioners might work with the clients and family members independently in the early stage, while conjoint sessions would be initiated in the later stage of the treatment. In the quote below, a practitioner described the rationale for this specific arrangement:

*I usually meet with the client and family members separately for rapport-building in the initial sessions. It is easier to gain trust and engage with adolescents when they feel safe to share their feelings and struggles in a private environment. Depending on different cases, I would arrange for parents to attend the session and provide space for them to negotiate and compromise with their children.*

(Participant 4 )

There were also times when the practitioner invited family members to participate in the treatment process via multiple formats, such as some relationship-building activities. A variety of options were offered to accommodate specific family circumstances in order to maximize the extent of family involvement as much as possible (*n* = 5).

## 4. Discussion

The present study adds to the previous literature by using qualitative methodology to examine the usefulness of family involvement in IA treatment with adolescents from the practitioners’ perspective. The first theme that emerged from the data highlighted the importance of family-based intervention. While the previous literature, such as that by King et al. [24], generally supports the view that treatment for adolescent IA should involve family members, little is known about their specific roles in the process. Results from the present study shed light on some directions of involving parents in IA intervention to improve treatment effectiveness. Through providing psychoeducation and individual counseling, practitioners not only discussed different constructive parenting strategies and communication skills with family members, but also reframed their experiences with their children’s IA behaviors by helping them to understand their unfulfilled needs and modifying their parenting roles and expectations. The result corresponds to a qualitative study on IA treatment considerations that the process of re-educating and empowering parents to set appropriate boundaries and expectations is essential for successful intervention outcomes [25]. This finding also aligns with the protective factors of IA, in which healthy parenting practices involving appropriate parental monitoring behaviors and strong emotional support are associated with a lower risk of IA occurrence for adolescents [6,8,26]. It is noteworthy that family members’ lack of emotional readiness and motivation could pose potential challenges. Parents may be preoccupied with their own mental health struggles or find discussing their personal history and early childhood experience irrelevant, and even offensive. Clinicians addressing their emotional needs and working through the denial phase could help family members develop an alternative perspective on the situation.

The second theme concerned the shift in intervention orientation from individual-focused to relationship- and family-focused. It was found that therapeutic alliances played a critical role in involving clients and family members in treatment sessions. This finding is consistent with previous literature that expounded that trustworthy therapeutic alliances not only constitute the basis of counseling, but also facilitate behavioral change for adults and adolescent clients in family and individual therapy [27]. Conjoint therapy sessions were one of the prominent components of family involvement. Practitioners facilitated positive communication and emotional flow between clients and family members, which set the stage for negotiation and compromise regarding acceptable Internet use. This finding coincides with results from previous research, such as Liu et al. [18] and Zhong et al. [16], that found that strengthened family cohesion and dynamics have profound effects on ameliorating IA behaviors. In accordance with recently published qualitative research on parents’ perspectives in gaming disorder interventions, counselors who put adequate emphasis on addressing family tensions and mediating turbulent parent–child relationships manage to expedite the treatment process [28]. Therefore, it is suggested that family involvement in IA treatment should encompass enhancing communication proficiency and fostering positive family interactions and functionality. Putting the results into cultural context and considering the traditional Chinese collective core family values, family involvement in the treatment process addresses the entire family as a unit [8,29]. This fits with the findings from a Singapore study that active parental participation in intervention programs is an effective, culturally relevant solution in the Asian context [28].

The third theme emphasized the need for individualized services and treatments since adolescents with IA are not a homogeneous group. Clinicians in this study did not merely report the use of an array of therapeutic approaches, but also suggested tailored treatment goals and duration, which is consistent with the findings of previous studies [20,30]. Despite the conclusive positive impacts of family involvement in the treatment process, the degree and arrangement of family involvement in IA treatment varied case-by-case. Special consideration of the parent–child relationship is warranted. When hostile and negative family interactions exist, involving family members in the treatment may be inappropriate and result in a disservice to the client and family. This finding corroborates with the existing literature in that caution is required due to the complicated family dynamic when modifying familial risk and protective factors of IA through a family-focused approach [9]. This implies that practitioners need to use their professional judgment to decide whether family-orientated interventions would be the optimal treatment for their clients. An extensive analysis of family-of-origin problems and communication patterns may be required in order to structure the intervention based on family circumstances and resources. For instance, a qualitative case study suggested that family therapy is imperative to ameliorate risks involved with IA for young adults suffering from interpersonal problems when the therapist is aware of the relationship between the characteristics of the family of origin and the presenting IA symptoms [31]. To address the classic question of, “What treatment, by whom, is most effective for this individual with that specific problem, and under which set of circumstances?” posed by Gordon Paul [32], further dedicated examination of potential subgroups of families and adolescents sharing similar characteristics within groups but different across groups will assist clinicians to tailor intervention or engagement strategies.

### 4.1. Limitations

The study’s findings should be interpreted in view of its limitations. One of the limitations is the study’s small sample size. As there are a limited number of professionals specialized in treating IA, recruitment of participants was difficult. The study was conducted during the peak of the COVID-19 pandemic, which further posed challenges to the recruitment process. Secondly, as the results of this study were derived from data collected in Hong Kong, the findings may not be generalized to other cultural contexts. Thirdly, the themes generated from the interviews are based primarily on the views of the participants, who were predominantly social workers. Other health professionals working with adolescents with IA, such as psychiatrists, pediatricians, and nurses, may have different views or treatment approaches when tackling the issue. Despite these limitations, this study gathered valuable views of professionals working with adolescents with IA, which provided future directions for practice and research.

### 4.2. Implications

Previous reviews of IA interventions show that previous interventions tended to take an individual-based approach, and only a small number of interventions involved family members [33,34]. Based on this study’s findings, involvement of family members in IA treatment for adolescents is recommended. Parents serve as an active agent in the intervention process, such as in learning how to cope with own emotional distress arising from the IA issue and ways to support the adolescent. Practitioners should first assess the stage of change of both the family members and the adolescent with IA and carefully consider whether and in which stage of treatment is family involvement most suitable. In the case where family conflicts may be a contributing factor to IA, special care and more in-depth groundwork is needed before engaging the family members in supporting the adolescents. Furthermore, a treatment approach that moves away from just treating the adolescent with IA and towards a relational approach is recommended. Specifically, both individual and family work are likely to be desirable. Treatment may begin with individual-focused interventions that can help address the individual needs of the adolescent with IA and the individual family members, and for the therapist to build a positive working relationship with each member. With the foundation of a positive working relationship and individuals’ needs being met, family-focused intervention will help address ways to modify the family environment and dynamics to better support the adolescent, to repair ruptures, and to reconnect family relationships.

In terms of research, additional quantitative research is needed to verify findings of this study. In particular, future studies on family-based intervention for adolescent IA, such as examining whether inclusion of both individual and family work is a promising approach and whether such an approach is applicable in different cultural contexts, are warranted. Given the heterogenous nature of families and individuals suffering from IA, research investigating potential subgroups of parent–adolescent dyads will provide useful insights into development of tailored interventions for the different subgroups.

## 5. Conclusions

This study highlights the importance and considerations of family involvement in IA treatment for adolescents in the Chinese context. Practitioners working with adolescents with IA should attend to family-related factors when creating treatment plans for parental participation. With meticulous arrangement and an optimal degree of involvement, family-focused intervention that involves both individual and family work is likely to be effective in facilitating the treatment process and promoting positive treatment outcomes through fostering effective communication and promoting family functioning. The findings of this study warrant further quantitative investigation into effective family-based treatment approaches to address IA in different cultural contexts.

## Figures and Tables

**Table 1:** Interview Questions.

Discussion Area	Questions
Experience in providing IA intervention to adolescents	What are your treatment goals/intervention orientations for adolescents with IA? Would you include family members in the treatment process? What would be some suitable and unsuitable circumstances for family involvement in the treatment process? What is your approach/arrangement when family members participate in the treatment? How do age and gender of adolescents affect your IA treatment design? How would you decide when to terminate treatment?
Effective IA intervention components	Based on your previous cases, what are the roles of family members in the treatment process? Thinking of a specific case that you worked with, what were the roles of the family members? What did you do in the treatment process? What was the treatment outcome? What do you think are the most important elements that contribute to the effectiveness of IA treatment? Why? What are some factors that enhance the treatment outcomes when family members are involved in the process? What are the difficulties/challenges of family participation in IA treatment?

**Table 2:** Demographic information of the participants.

Participant	Age	Sex	Highest Education Level	Profession	Social Service Field	Self-Identified Therapeutic Approach	Clinical Experience in Working with Youth and Family (Years)	Clinical Experience in Working with Adolescents with IA (Years)
1	36–40	F	Master’s degree	Counsellor	Addiction Counselling Service	Expressive arts	10	7
2	51 or above	F	Master’s degree	Registered Social Worker	Youth Services	Narrative Practice/Narrative Therapy	28	9
3	31–35	M	Master’s degree	Registered Social Worker	Family Services	Narrative, Strength-based	8	6.5
4	31–35	F	Bachelor’s degree	Registered Social Worker	Addiction Counselling Service	Narrative, Satir	9	7
5	26–30	F	Master’s degree	Counselling Psychologist	Addiction Counselling Service	Client-centered Therapy, Cognitive Behavioral Therapy, psychodynamic	1	1
6	26–30	M	Bachelor’s degree	Registered Social Worker	Addiction Counselling Service	Satir Family Therapy	4	1
7	31–35	M	Bachelor’s degree	Registered Social Worker	Addiction Counselling Service	Multiple intervention approaches	10	3
8	36–40	M	Master’s degree	Counsellor	Addiction Counselling Service	Cognitive Behavioral Therapy	11	11
9	26–30	M	Master’s degree	Registered Social Worker	Addiction Counselling Service	Multiple intervention approaches	2	2
10	36–40	F	Master’s degree	Registered Social Worker	Addiction Counselling Service	Motivational interviewing, Cognitive Behavioral Therapy	11	11

**Table 3:** Phases of thematic analysis.

Phase of Thematic Analysis	Detailed Account of Process
Phase 1: Familiarizing with the data	Read and re-read the data, get immersed and familiar with its content
Phase 2: Coding	Begin line-by-line coding the entire dataset, generate codes to capture important features of the data that potentially answer the research questions, develop the initial codebook, collate all the codes and relevant data extracts for later stages of analysis, discuss, and resolve discrepancies in coding
Phase 3: Generating initial themes	Combine and merge similar codes, sort out the high-frequency codes and conceptualize into tentative themes by identifying significant broader patterns of meaning, collate relevant data to each candidate theme, develop hierarchies of concepts, take notes for the generation of latent themes
Phase 4: Reviewing themes	Check the tentative themes against the dataset, refine the themes to ensure each reflects the pattern of shared meaning underpinned by a central concept/idea
Phase 5: Defining and naming themes	Iron out the scope and focus of each theme, resolve the discrepancies in theme generation, decide on an informative name for each theme
Phase 6: Writing up	Describe the process of coding and analysis, report on methodological and analytical choices, write up findings supported by illustrative quotes

**Table 4:** Themes and illustrative quotes from thematic analysis.

Theme	Number of Participants Mentioned	Illustrative Quotes
Family involvement in IA intervention is challenging yet important	8	Example 1: In many IA cases, the adolescent is not my sole client. Instead, I typically provide services to the entire family and tend to involve family members throughout the treatment process (Participant 7). Example 2: I believe family involvement in the treatment process can bring positive outcomes. While the situation may become more complicated and difficult with some malfunctioning families, it is important for the parents to adjust and participate in the intervention (Participant 4).
Shifting the focus from the adolescent to the relationship	6	Example 3: When I invite parents to join the counseling session, my aim focuses on improving their parent–child relationship, which I believe would be helpful in reducing the client’s IA behaviors (Participant 2). Example 4: Parent–child relationship is usually a more pronounced problem in comparison to IA when family members are involved in the treatment process. Rebuilding a healthy relationship and fostering constructive communication are my primary goals (Participant 9).
Provision of individualized services and intervention to address the heterogeneous nature of cases	8	Example 5: I do not have a standardized approach or therapeutic orientation in handling IA cases. Depending on the age and needs of the client and family members, as well as the goals of the intervention, I adopt a mix of different approaches. For instance, motivational interviewing is more suitable for encouraging engagement while cognitive-behavioral therapy can help identify distorted thoughts and initiate behavioral change (Participant 8). Example 6: The client’s needs and treatment goals are my major considerations when deciding whether family involvement is beneficial to the intervention process. Family relationship is another critical factor that determines if conjoint therapy sessions would be appropriate (Participant 3).

## Data Availability

Not applicable.
